# Aortic sinus aneurysm invading ventricular septum and dissection caused by Behcet’s disease: a case report and literature review

**DOI:** 10.1186/s12872-023-03420-7

**Published:** 2023-08-30

**Authors:** Pengjia Wu, Shaomei Yu, Jiashun Zeng, Lei Yang

**Affiliations:** Department of Rheumatology and Immunology, The Affiliated Hospital of Guizhou Medical University, Guiyang, 550004 China

**Keywords:** Behcet’s disease, Aortic sinus rupture, Intercalation of interventricular septum forms

## Abstract

Few case reports have mentioned the aortic sinus aneurysm invading ventricular septum and dissection caused by Behcet’s disease. Here, we reported a 36-year-old male patient with an aortic sinus aneurysm invading the ventricular septum and dissection caused by Behcet’s disease, who manifested as recurrent chest tightness and shortness of breath. Cardiac ultrasound showed the rupture of the right aortic sinus and the formation of ventricular septal dissection. Ascending aortic valve prosthesis replacement, mitral valvuloplasty with ring implantation and tricuspid valvuloplasty were performed. Postoperatively, he was treated with hormones, hydroxychloroquine sulfate, mycophenolate mofetil tablets, thalidomide and warfarin, and his symptoms were relieved. This is a rare case easily being misdiagnosed and missed, early diagnosis and in-time treatment are crucial to avoid surgical complications. The diagnostic and therapeutic approaches of this patient were reported and related literature was reviewed in this case report.

Behcet’s disease (BD), also known as oral-oculo-genital syndrome, is a chronic systemic vascular inflammatory disorder characterized by recurrent oral ulcers, genital ulcers, ocular manifestations (e.g., uveitis, conjunctivitis), and other systemic involvement. It is more common in Mediterranean coastal countries such as Greece, Middle Eastern countries such as Turkey, and East Asian countries such as China, Korea, and Japan, which roughly coincide with the ancient Silk Road, so it is also known as “Silk Road” disease. When the heart is involved, it is called cardiac Behcet’s disease (CBD), which mainly includes heart valve disease, conduction system disorders, endocardial fibrosis, myocarditis, pericarditis and acute myocardial infarction [1]. There are few reports of aortic sinus aneurysm ruptured into the interventricular septum to form dissection caused by cardiac Behcet’s disease, which is easily misdiagnosed and missed in clinical practice. Here we report the diagnosis and treatment of a patient with aortic sinus aneurysm ruptured into the ventricular septum to form a dissection caused by cardiac Behcet’s disease, and analyze the characteristics and treatment of the disease by reviewing the relevant literature.

## Case summary

The patient (male, 36-year-old) was admitted due to “chest distress and shortness of breath for more than 2 months” in March 2019. The patient complained of previous recurrent oral ulcers and external genital ulcers for more than 10 years, and his mother and 1 younger brother and 1 sister also had a history of recurrent oral ulcers. He had a history of hypertension for more than 10 years, and received “left carotid artery stent implantation” in another hospital due to “left carotid artery aneurysm” in 2016. Due to the pseudoaneurysm formation at the puncture site of left femoral artery, this patient received “left femoral artery stent implantation” again in 2016. After the operation, he was given Aspirin for anti-platelet aggregation for a long time.

**Fig. 1:**
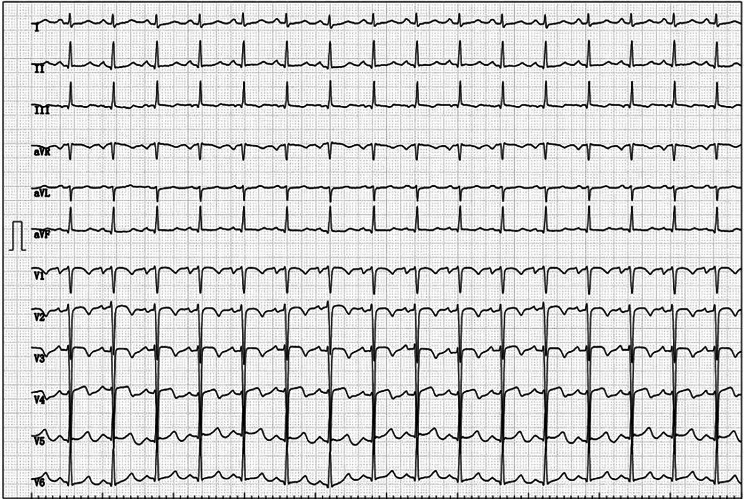
Electrocardiogram of this patient. Poor V1-V4R wave increment in the chest lead, arch back elevation in the V2V3ST segment and inversion of the V3V4T wave

**Fig. 2:**
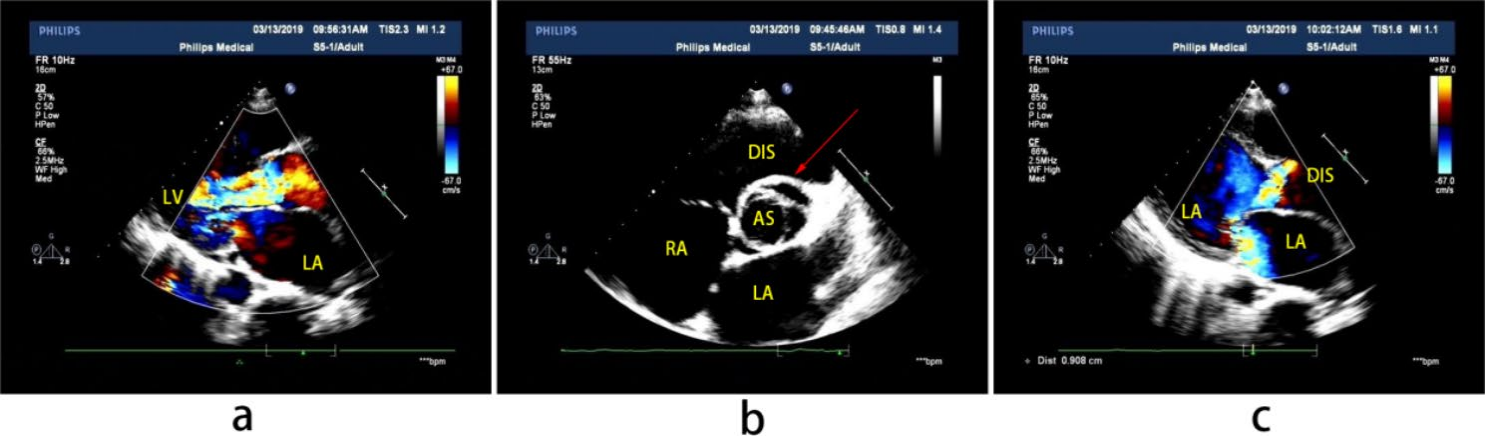
Pre-operation echocardiography of this patient. **(a)** Formation of interventricular septal dissection (yellow arrow), entry into left ventricle behind the anechoic area in the upper part of the interventricular septum. **(b)** Rupture of right aortic sinus, blood flowing from the right sinus part to the interventricular septal dissection and then to the left ventricle. **(c)** Mitral regurgitation. LV, left ventricle; LA, left atrium; RA, right atrium; AS, aortic sinus; DIS, dissection

In January 2019, the patient experienced chest distress and shortness of breath occurred without obvious inducement, and his symptoms were aggravated after activities, accompanied by thoracic pain, and occasional orthopnea at night, without fever, cough or expectoration. The patient visited another hospital, he was diagnosed with “pulmonary infection, rheumatic heart disease”. Then he was admitted to our hospital for further “heart valve replacement” in March 2019. The physical examination revealed that the jugular vein was filled, tremor was palpable in the auscultatory area of the aortic valve, the heart rate was regular, grade 4/6 systolic blowing murmur could be heard in the auscultatory area of the aorta, and grade 3/6 systolic blowing murmur could be heard in the auscultatory area of the mitral and tricuspid valves and showed small ulcers in the left buccal mucosa and scattered acne-like nodules in the chest and back. Laboratory examinations showed as follows: myocardial markers: high-sensitivity troponin 0.022 ng/ml, brain natriuretic peptide 5435 pg/ml, C-reactive protein 6.11 mg/l, erythrocyte sedimentation rate 15 mm/h. Electrocardiogram revealed a sinus rhythm of 99 beats per minute, poor V1-V4R wave increment in the chest lead, and ST-T changes (arch back elevation in the V2V3ST segment and inversion of the V3V4T wave) (Fig. 1). Echocardiography results showed the aortic sinus rupture (right sinus) with interventricular septal dissection, a large amount of shunt between the aortic sinus and interventricular septum, slightly coiled and moderate mitral valve cusp, tricuspid valve enlargement (a small amount), left heart and right atrium enlargement (Fig. 2). Enhanced aortic CTA showed the arc-shaped low-density shadow of ascending aorta, considering pulsation-related artifact; aortic CTA showed no definite sign of aortic dissection.The pathergy test was negative and no ocular lesions.

**Fig. 3:**
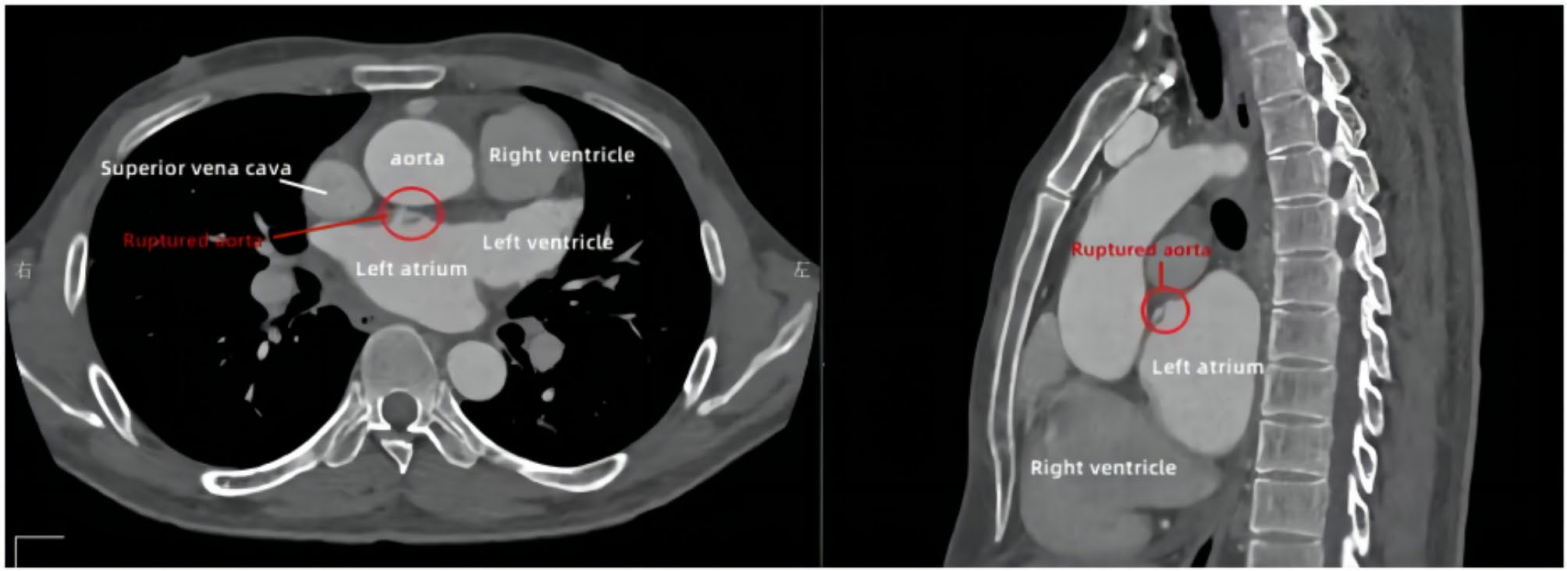
Enlargement of left atrium and left ventricle, widening and rupture of aortic sinus, irregular shape of aortic valve

According to the international standard score for Behcet’s disease established in 2013 [2], the patient was diagnosed with cardiac Behcet’s disease, aortic sinus rupture and ventricular septal dissection. Considering that the patient had severe vascular disease and complicated conditions, and underwent surgery, the patient was given Prednisone Acetate 35 mg/d, Azathioprine 25 mg bid and Aspirin 0.1 g/d for treatment.The patient did not improve the cardiac CTA examination initially due to economic reasons. After 2 months of treatment with prednisone acetate and azathioprine, the improvement of cardiac CTA still indicated the rupture of the aortic sinus and the formation of interventricular septum dissection (Fig. 3).

**Fig. 4:**
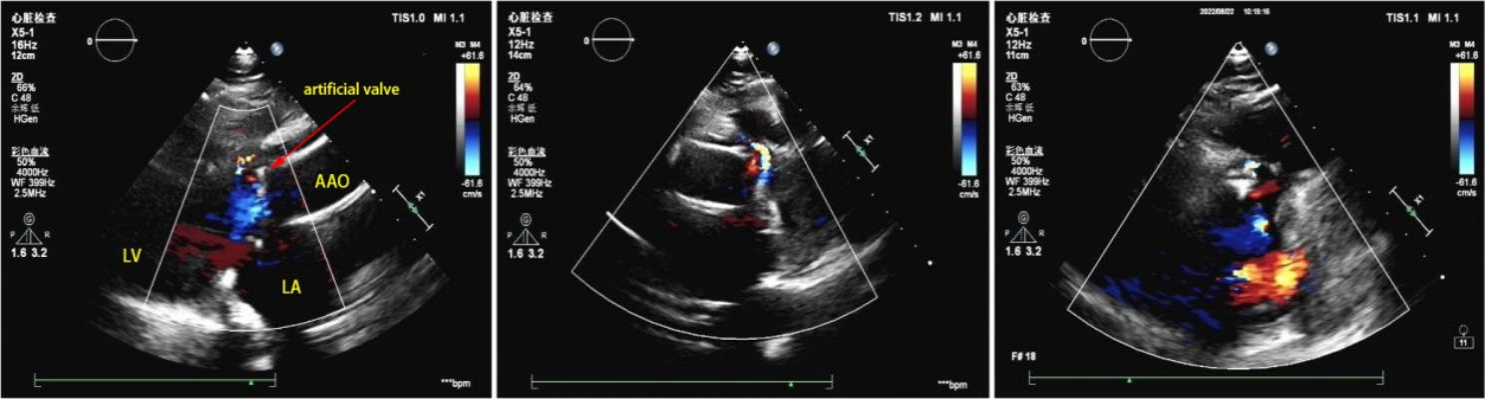
Post-operation echocardiography of the patient. A 4-mm gap between the artificial ascending aorta and the autologous vessel could be detected. The aortic valve was an artificial mechanical valve (yellow arrow) with a fixed mechanical annulus at approximately 12 o’clock, with a 3-mm spacing between the artificial and autologous annuli. The mitral valve was seen to have a strong echogenic forming ring. LV, left ventricle; LA, left atrium; AAO, ascending aorta

Then followed by “Ascending aortic valve prosthesis replacement + mitral valvuloplasty with ring implantation + tricuspid valvuloplasty” in June 2019. After the operation, he was given Prednisone Acetate 35 mg/d, Hydroxychloroquine Sulfate tablets 0.2 g bid, Mycophenolate Mofetil tablets 0.5 g bid, Thalidomide tablets 25 mg/d and Warfarin 3.75 mg/d for treatment. During this period, the patient revisited regularly. In February 2022, his cardiac ultrasound during follow-up showed “Behcet’s disease, Bentall + post mitral valvuloplasty, normal mechanical flap function” (Fig. 4). Prednisone Acetate had been gradually reduced to 5 mg/d, Warfarin 3.75 mg/d was still used for anticoagulation. Till May 2022, the patient’s condition is stable, without chest tightness, shortness of breath under general activities, skin ulcers or ulcers at other sites.

## Discussion

Behcet’s disease is a chronic relapsing systemic vasculitis that can affect almost all organs and systems of the body with variable manifestations. It has been reported that the proportion of patients with Behcet’s disease with cardiovascular involvement is about 7 − 46% and the proportion of patients with Behcet’s disease with significant vascular involvement is about 20% [3].

In this case, the patient mainly presented with the formation of an aortic sinus aneurysm, which ruptured into the interventricular septum to form a dissection, and was associated with aortic insufficiency. Causes of aortic sinus aneurysm formation include atherosclerosis, syphilis, Marfan syndrome, trauma, and Behcet’s disease, etc. [4]. Some studies have reported that the formation of aortic sinus aneurysm is the main cause of death in patients with Behcet’s disease [5]. In our case, echocardiography showed the rupture of the aortic sinus (right sinus) with interventricular septal dissection, so was Behcet’s disease responsible for the formation and rupture of his aortic sinus aneurysm? Through careful inquiry of medical history, the patient complained of previous recurrent oral ulcers and external genital ulcers for more than 10 years, and his mother and 1 younger brother and 1 sister had a history of recurrent oral ulcers, scattered acneiform nodules on the chest and back, and small ulcers on the left buccal mucosa. Combined with his medical history, the patient had previous recurrent hemangiomas, underwent “left carotid artery stenting” more than 3 years ago due to “left carotid artery aneurysm”, had multiple postoperative arterial fistulas, and underwent “left femoral artery stenting” again due to pseudoaneurysm formation at the left femoral artery puncture site, which provided clues for diagnosis and differential diagnosis. If these symptoms were missed or neglected in clinical practice, it may cause misdiagnosis and delay the best time for treatment of patients.

Till now, only a few case reports have been published on the interventricular septal dissection caused by Behcet’s disease. Chen et al. [6] reported a 44-year-old male patient who has right sinus of Valsalva aneurysm spontaneously dissecting into the interventricular septum, transthoracic echocardiography revealed a 5 mm perforation of right SoV aneurysm that had subsequently dissected into the interventricular septal and formed massive cystic-like mass, with an uneven-echo right coronary cusp and mild-to-moderate aortic regurgitation. They mentioned that alertness of the diverse echocardiographic features of cardiac Behcet’s disease is crucial for early diagnosis and long-term prognosis. Consistently, our patient showed typical abnormalities such as aortic sinus rupture with interventricular septal dissection, and a large amount of shunt between the aortic sinus and interventricular septum detected by echocardiography, providing important image evidence to diagnose. Similar case reports about interventricular septal dissection caused by Behçet’s Disease were also reported [7–9]. Yu et al. [9]. indicated their findings in Echocardiographic features of interventricular septal dissection in patients with Behçet’s Disease, which include echo-free space in the interventricular septal segment or basal to middle segment, dilatation in the diastole and contraction in systole, and abnormal turbulent blood flow in the heart. Here, we also noticed the blood flowing from the right sinus part to the interventricular septal dissection and then to the left ventricle, accompanied by mitral regurgitation. However, Yu et al. [9]first used methylprednisolone and thalidomide to control BD in their case, and the surgical treatment has been performed after the BD remission. Similar consideration also reported that immediate surgical repair might not be a good choice for these patients with BD, the surgery should be carried out after immunosuppressive therapy and BD remission, which helps reduce the postoperative complications [10, 11]. In our case report, due to the severe vascular disease and complicated conditions of this patient, he received perioperative immunosuppressive treatment and accepted the surgery, and his long-term follow-up showed a favorable outcome. Collectively, the therapeutic strategy of BD patients accompanied by interventricular septal dissection should be formulated based on individual characteristics, personalized treatment may help BD patients achieve the optimal prognosis.

At present, the etiology of Behcet’s disease is still not clear [12], and some studies suggest that viral, bacterial, genetic, environmental, toxic and immune factors all play an important role in the pathogenesis [13]. However, the hypothesis of greatest concern is that certain viruses, bacteria, or other environmental factors and/or autoantigens such as heat shock proteins induce an autoimmune response in the genetic population, which in turn causes vasculitis [14]. Ulusan Z et al. [15] showed that Behcet’s disease histopathology was non-specific vasculitis, and its monocyte and neutrophil infiltration, endothelial cell proliferation, destruction of the elastic layer, fibrinoid necrosis, and thrombosis were pathological evidence of the disease at the tissue level.

In patients with Behcet’s disease, 22% and 3%, respectively, have a history of large vessel veins or arteries [16], and these lesions often appear as complications in the late stages of the disease. Our patient suffered from left carotid artery aneurysm 3 years ago, and recurrent pseudoaneurysm occurred at the puncture site after interventional therapy. As early as 1981, James DG et al. [5] reported that angiographic arterial puncture, cardiopulmonary bypass or aneurysm bypass surgery may lead to vascular interference or further formation of pseudoaneurysm at the anastomosis site. Aneurysms can appear in the thoracic and abdominal aorta, superior mesenteric arteries, and arteries of the extremities, and these aneurysms have the potential for leakage, rupture, or thrombosis, and thrombosed aneurysms may be the source of peripheral embolism, and aortic aneurysmal dilatation was detected in 48% of the patients in their study [5]. While previous studies have also reported that cases of aortic aneurysmal dilatation are about 5 − 30% [17, 18]. Aortic root dilatation may be an early lesion of the aneurysm. Aneurysms are the leading cause of death because of the risk of rupture. Especially in patients with aortic root dilatation, echocardiography can be performed regularly before severe aneurysms and complications occur [5]. Some investigators believe that compared with aneurysms caused by atherosclerosis, Behcet’s disease-related aneurysms are at risk of rupture, and rupture is not related to aneurysm size. Therefore, they recommend that aggressive and invasive methods should be adopted once the diagnosis is confirmed [19]. However, others have also shown that while aneurysms are at risk of rupture, the incidence of complications after surgical intervention is also high [20–22]. Therefore, Liu Q et al. [22] suggested that surgical intervention may not be necessary for patients with small, intact saccular aneurysms. There is no consensus on whether to perform bypass surgery or endovascular stent implantation for peripheral aneurysms caused by Behcet’s disease [19, 21–24]. To prevent thromboembolic complications after stenting in patients with peripheral aneurysms, anticoagulant therapy should also be given to those complicated by occlusive arterial disease after aneurysm repair [22].

Studies have reported that aortic sinus aneurysm may cause complications, such as right ventricular outflow tract obstruction, coronary artery occlusion, aortic valve and congestive heart failure, if left untreated, so surgical treatment needs to be considered [25, 26]. In our case, the patient had a ruptured aortic sinus aneurysm caused by Behcet’s disease and ruptured into the interventricular septum to form a dissection, combined with aortic insufficiency. The analysis showed that the reason may be that non-specific inflammation caused the destruction of aortic sinus structure, followed by intimal tear under the impact of high-pressure blood flow, and extended to the endocardium with existing inflammatory changes [27]. The patient had surgical indications. The patient was treated with glucocorticoids and immunosuppressive agents during and after the perioperative period, and was given anticoagulant therapy after the operation. The disease was fairly controlled, without the tendency of aneurysm recurrence, which was similar to previous reports [15, 22, 28].

Behcet’s disease is a collagen vascular disease with high cardiac involvement, but it is rare to cause aortic sinus aneurysm rupture and rupture into the interventricular septum to form a dissection in clinical practice. The disease is serious and has a high risk of death. It is easy to miss the diagnosis and misdiagnosis. Early identification is essential for the selection of treatment options, avoiding or reducing surgical complications and the long-term prognosis of patients. Therefore, close cardiac detection for patients with Behcet’s disease is very important.

## Data Availability

All data supporting the conclusions are presented in the manuscript.
